# CE–RAA–CRISPR Assay: A Rapid and Sensitive Method for Detecting *Vibrio parahaemolyticus* in Seafood

**DOI:** 10.3390/foods11121681

**Published:** 2022-06-08

**Authors:** Xinrui Lv, Weiwei Cao, Huang Zhang, Yilin Zhang, Lei Shi, Lei Ye

**Affiliations:** 1 Institute of Food Safety and Nutrition, Jinan University, Guangzhou 510632, China; lxrui_1995@163.com (X.L.); 18680288520@163.com (H.Z.); stzhyl@163.com (Y.Z.); leishi88@hotmail.com (L.S.); 2 College of Food and Bioengineering, Guangdong Polytechnic of Science and Trade, Guangzhou 510640, China; weiwei09201029@163.com

**Keywords:** recombinase-aided amplification, CRISPR/Cas12a, chemical, fluorescence detection, *Vibrio parahaemolyticus*

## Abstract

*Vibrio parahaemolyticus* is one of the major pathogenic *Vibrio* species that contaminate seafood. Rapid and accurate detection is crucial for avoiding foodborne diseases caused by pathogens and is important for food safety management and mariculture. In this study, we established a system that combines chemically enhanced clustered regularly interspaced short palindromic repeats (CRISPR) and recombinase-aided amplification (RAA) (CE–RAA–CRISPR) for detecting *V. parahaemolyticus* in seafood. The method combines RAA with CRISPR-associated protein 12a (Cas12a) for rapid detection in a one-pot reaction, effectively reducing the risk of aerosol contamination during DNA amplifier transfer. We optimized the primers for *V. parahaemolyticus*, determined the optimal crRNA/Cas12a ratio, and demonstrated that chemical additives (bovine serum albumin and L-proline) could enhance the detection capacity of Cas12a. The limit of detection (at optimal conditions) was as low as 6.7 × 10^1^ CFU/mL in pure cultures and 7.3 × 10^1^ CFU/g in shrimp. Moreover, this method exhibited no cross-reactivity with other microbial pathogens. The CE–RAA–CRISPR assay was compared with the quantitative polymerase chain reaction assay using actual food samples, and it showed 100% diagnostic agreement.

## 1. Introduction

*Vibrio parahaemolyticus* (*V. parahaemolyticus*) is a common Gram-negative foodborne pathogenic bacterium. It is a halophilic marine bacterium that commensally exists in various seafoods, including fish, shrimp, and shellfish [1], and is one of the leading causes of seafood poisoning [2]. As a zoonotic pathogen, *V. parahaemolyticus* causes gastrointestinal illness characterized by watery diarrhea, nausea, vomiting, abdominal cramps, and headache. In severe cases, it may result in septicemia and fatal diseases [3]. Consumption of raw, parboiled, or contaminated seafood is one of the most common causes of food poisoning in humans [4]. In the aquaculture industry, *V. parahaemolyticus* can also cause acute or severe gastroenteritis infections in aquatic animals, resulting in huge economic losses [5]. Therefore, developing technologies for the sensitive and rapid detection of this pathogen is vital to ensure the safety of seafood.

Currently, conventional culture-based methods are considered the gold standard for the detection of *V. parahaemolyticus*. However, these methods are time-consuming and labor-intensive, usually requiring two to three days to obtain results [6], and the methods may underestimate the microbial population size [7]. Thus, polymerase chain reaction (PCR) methods, including quantitative PCR (qPCR) and droplet digital PCR (ddPCR), have been widely used as alternative methods for the rapid detection of *V. parahaemolyticus* [8,9]. However, these methods are inconvenient for use in grassroots laboratories and on-site applications, as they require expensive instruments, complex detection procedures, and skilled operators [10,11].

To overcome these limitations, some isothermal amplification techniques such as loop-mediated isothermal amplification (LAMP) and recombinase-aided amplification (RAA) have been developed. These techniques are suitable for environments lacking infrastructure, expensive instruments, and technical expertise. Compared with LAMP, RAA is characterized by relatively fast reaction (<30 min) and operates at low temperature (37–42 °C) [12,13]. Although RAA is more portable than the PCR method, further improvement in sensitivity is required for *V. parahaemolyticus* detection.

In recent years, clustered regularly interspaced short palindromic repeats (CRISPR) and CRISPR-associated (Cas) enzymes have not only revolutionized the field of genome editing but also shown remarkable potential in the field of nucleic acid detection [14,15,16]. CRISPR/Cas12a exhibits trans-cleavage activity for nonspecific single-stranded DNA (ssDNA). Cas12a combines with crRNA to form a Cas12a-crRNA complex, which then specifically recognizes the DNA targets to form a ternary complex (Cas12a/crRNA/DNA targets). The trans-cleavage activity of the Cas12a protein for ssDNA is triggered by the specific recognition of the target DNA. Thus, through the fluorophore and quencher labeling of the ssDNA, the content of the target gene can be converted into a fluorescent signal [17,18,19]. The CRISPR system has been combined with the RAA assay to detect various foodborne pathogens, including *Listeria monocytogenes* [20], *Salmonella* [21], and spoilage bacteria [22].

In previous studies, researchers have focused on optimizing the composition of the system (e.g., Cas nuclease, reporter, and Mg^2+^), adjusting the stoichiometry between the Cas protein and crRNA, or modifying the crRNA to improve detection [23,24]. In addition, several researchers have reported that the reaction efficiency can be further enhanced through chemical addition. For example, the chemically enhanced CRISPR detection method called CECRID was applied for detection [25]. However, its effectiveness in the detection of *V. parahaemolyticus* in raw shrimp samples has rarely been investigated; therefore, the factors affecting its sensitivity are unclear. Moreover, the method requires the transfer of amplification products, which can cause aerosol contamination.

**Figure 1:**
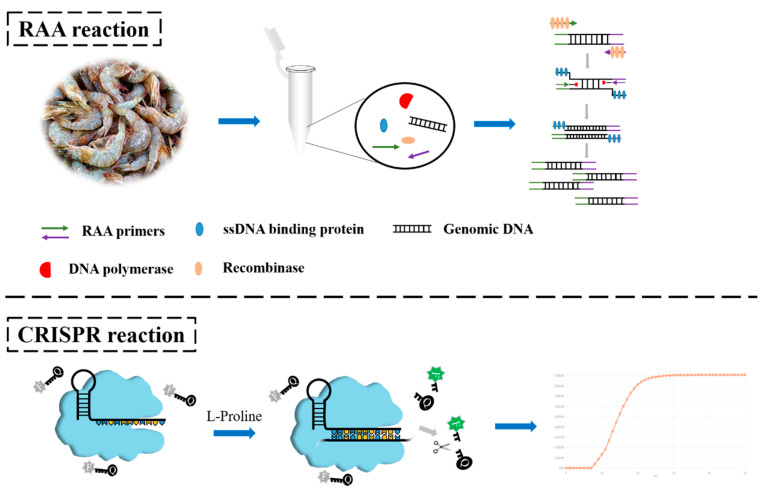
Schematic illustration of the principle of CE–RAA–CRISPR.

To improve the convenience of existing tools and overcome the limitations of *V. parahaemolyticus* detection, in the present study, we designed a novel system that combines chemically enhanced CRISPR detection and RAA (CE–RAA–CRISPR). In this system, the RAA reagent is placed at the centrifuge tube bottom for amplification. After amplification, the CRISPR reagent is placed at the top of the centrifuge tube, and the two reagents are mixed via centrifugation, which triggers the CRISPR assay. We optimized the system composition and evaluated the effects of the addition of several chemicals on the detection signal. Finally, a sensitive, rapid, and accurate CE–RAA–CRISPR assay was obtained for detecting *V. parahaemolyticus* in shrimp samples (Figure 1). This method provides new ideas for the detection of foodborne pathogens while reducing amplicon contamination.

## 2. Materials and Methods

### 2.1. Bacteria Culture and Genomic DNA Extraction

The bacterial strains used in this study are listed in Table 1. All of the *Vibrio* species strains were inoculated in a sterile 3% NaCl alkaline peptone water (APW, Huankai Microbial Sci. & Tech. Co., Ltd. [HM], Guangzhou, China) medium, and the rest of the strains were incubated in a Luria-Bertani (LB, HM) broth at 37 °C for 7 h. Cells were serially diluted using sterile 0.85% (*w*/*w*) NaCl to obtain 10-fold serial dilutions. Furthermore, 3% NaCl tryptone soy agar (3% NaCl TSA, HM) was used for enumerating the viable count of *V. parahaemolyticus*. DNA extraction was performed using a bacterial genomic DNA extraction kit (Vazyme Biotech Co., Ltd., Nanjing, China) according to the manufacturer’s protocol. The extracted genomic DNA was stored at −20 °C until use.

### 2.2. Primer Design

Primers and probes (Table 2) were designed according to the conserved *tlh* gene sequence of *V. parahaemolyticus* using Primer Express 3.0.1 and synthesized by Sangon Biotech (Shanghai, China). Before the primers were used, their specificity was evaluated through a BLAST search using sequences in GenBank.

### 2.3. Standard RAA System

The RAA reaction was performed using an RAA nucleic acid amplification kit (Jiangsu Qitian Gene Biotechnology Co., Ltd., Wuxi, China) following the manufacturer’s instructions, but with slight modifications. Each 10 μL of reaction mixture contained 2 μL of template, 0.4 μL (10 μM) of each primer, 5 μL of RAA solution, 0.6 μL (280 mM) of magnesium acetate, and 1.6 μL of nuclease-free water. The mixture was incubated in a metal bath at 37 °C for 30 min.

### 2.4. Real-Time RAA and PCR

An RAA fluorescence detection kit (Jiangsu Qitian Gene Biotechnology Co., Ltd.) was used for real-time RAA (qRAA) assay optimization. This was carried out by adding 2 μL of genomic DNA, 0.42 μM of each primer, 0.12 μM of the probe, 14 mM of magnesium acetate, 10 μL of RAA buffer solution, and nuclease-free water up to 20 μL. The reaction tubes were placed on DHelix-Q5 (Guangzhou Double Helix Gene Technology Co., Guangzhou, China) at 37 °C for 30 min, and the fluorescence signal was recorded every 30 s.

qPCR was conducted following a previously published method, with modifications. The qPCR reaction mixture contained 10 μL of AceQ^®^Universal U+ Probe Master Mix V2 (Vazyme Biotech, Nanjing, China), genomic DNA template (2 μL), 0.4 μL of forward primer (10 μM), 0.4 μL of reverse primer (10 μM), 0.2 μL of probe (10 μM), and 7 μL of nuclease-free water. The reaction tubes were placed in an ABI QuantStudio6 Q6 system (ABI, USA) to collect fluorescence signals. The qPCR cycling conditions were as follows: 37 °C for 2 min to eliminate false-positive contamination, 5 min denaturation at 95 °C, followed by 45 cycles of 10 s denaturation at 95 °C, and 60 °C for 30 s for annealing-extension.

### 2.5. CE–RAA–CRISPR System

The LbaCas12a (Guangzhou Bio-Lifescsi Co., Ltd., Guangzhou, China) complex was preassembled as follows: 2 μL of 10× reaction buffer, 200 nM of LbaCas12a protein, 100 nM of crRNA, 500 nM of ssDNA-FQ reporter, 2 μL of bovine serum albumin (BSA, 1 mg/mL) solution, and 2 μL of L-proline (5 M), which we added to a 10 μL reaction system. For the CE–RAA–CRISPR assay, we first added 10 µL of liquid paraffin to the RAA reaction system to prevent aerosol pollution. After RAA, the LbaCas12a complex was added to the PCR tube cap and mixed via centrifugation. All primers were synthesized by Sangon Biotech (Shanghai, China). CrRNA and ssDNA were synthesized by Guangzhou Bio-Lifescsi Co., Ltd.

### 2.6. Evaluation of Limit of Detection (LOD) of qRAA, RAA–CRISPR, and CE–RAA–CRISPR in Pure Culture

The LODs of the qRAA, RAA–CRISPR, and CE–RAA–CRISPR assays were tested through gradient dilution of the *V. parahaemolyticus* suspension. The *V. parahaemolyticus* suspension was diluted with sterile 0.85% (*w*/*w*) NaCl in a ten-fold gradient, and then DNA was extracted as a template for qRAA, RAA–CRISPR, and CE–RAA–CRISPR assays. The remaining steps were similar to the process described above.

### 2.7. Evaluation of CE–RAA–CRISPR in Artificially Contaminated Shrimp

Commercially available raw shrimp samples were purchased from a local supermarket (Guangzhou, China). Prior to the start of the experiment, the absence of *V. parahaemolyticus* in the sample was determined through the qPCR method. A 25 g shrimp sample was placed in a sterile sampling bag, 225 mL of sterile 0.85% (*w*/*w*) NaCl was added, and the sample was manually ground with a grinding rod until a sample homogenate was formed. To evaluate the LOD of CE–RAA–CRISPR using artificially contaminated shrimp, 1 mL of different concentrations of *V. parahaemolyticus* (7.3 × 10^5^ to 7.3 × 10^1^ CFU/mL) was mixed with 9 mL of sample homogenate to prepare artificially contaminated shrimp samples with *V. parahaemolyticus* at concentrations of 7.3 × 10^4^ to 7.3 × 10^0^ CFU/g (no incubation). Then, 1 mL aliquots were collected at each gradient, and DNA was extracted according to the method described in Section 2.1. The obtained DNA was used as the template for the subsequent CE–RAA–CRISPR assay. Each food sample was tested twice through the CE–RAA–CRISPR assay, and the experiment was repeated three times.

### 2.8. Determination of Specificity

We used 22 bacterial strains, including 11 *V. parahaemolyticus* strains, 1 other *Vibrio* species, and 10 non-*V. parahaemolyticus* strains, to determine the specificity of the CE–RAA–CRISPR method. Genomic DNA from these strains was extracted as a template.

### 2.9. Comparison of CE–RAA–CRISPR with qPCR Assay on Real Samples

To test actual samples, 40 aquatic food samples were obtained from local markets (Guangdong Province, China), including 20 shrimp, 10 fish, and 10 clam samples. We added 225 mL of sterile APW per 25 g food sample in a sterile sampling bag, which we ground manually to form a homogenate sample, and then incubated at 37 °C for 3 h. Then, genomic DNA was extracted from 1 mL of the homogeneous solution and used as a template for CE–RAA–CRISPR and qPCR [7].

### 2.10. Data Analyses

The results are expressed as mean ± standard deviation. All statistical analyses were performed using R (Version 4.1.0, R core team). A *p* value of less than 0.05 was considered statistically significant. All graphs were constructed in Origin 2022.

## 3. Results

### 3.1. Optimization of Primers for RAA

**Figure 2:**
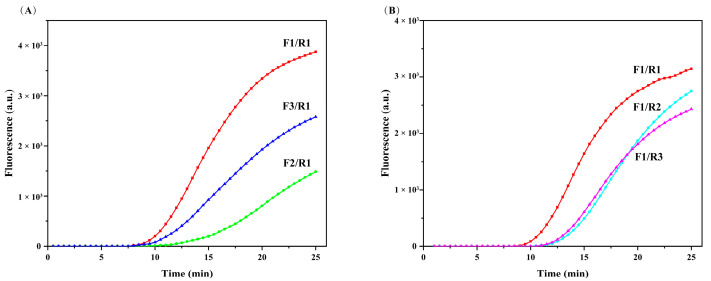
qRAA amplification results of the different primer combinations. Screening of upstream primer (**A**) and downstream primer (**B**).

The RAA efficiency is strongly influenced by the primer sequence. Therefore, we examined different primer combinations using the *V. parahaemolyticus tlh* gene as the target gene. Three upstream primers and three downstream primers were designed for screening, while a fluorescent probe was designed to bind to the primers for detection via qRAA. The downstream primer R1 was used in combination with the upstream primers in sequence. The best amplification efficiency was achieved when R1 was combined with upstream primer F1. When 1 × 10^6^ CFU/mL DNA was used as the template, the fluorescence signal started to appear after ~8 min. Among all primer combinations, the F1–R1 combination featured the earliest peak time and the highest endpoint fluorescence value. The other primer combinations showed slightly later peak and lower endpoint fluorescence than F1–R1 (Figure 2A). F1 was selected for screening with all reverse primers, and only R1 yielded the best results (Figure 2B). Therefore, the F1–R1 combination was considered the optimal primer pair.

### 3.2. Optimization of CRISPR Reaction

**Figure 3:**
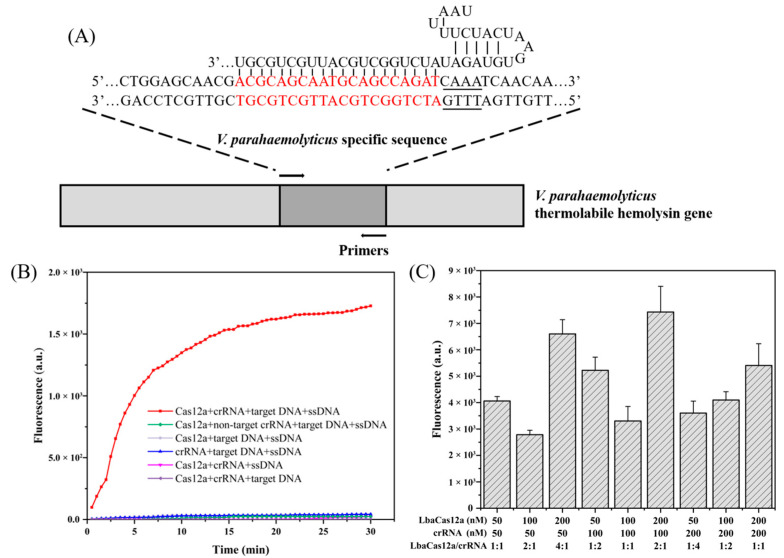
Feasibility analysis of RAA–CRISPR for nucleic acid detection. (**A**) Sequences of crRNA designs used for *V. parahaemolyticus* detection. (**B**) Feasibility of RAA–CRISPR for *V. parahaemolyticus* detection in fluorescence reporting. (**C**) Optimization of the Cas12a/crRNA concentration.

The crRNA–DNA target complementarity was determined according to the RAA amplification region (Figure 3A). The trans-cleavage activity of the Cas12a protein can only be activated when Cas12a/crRNA and the target are present. At this point, the ssDNA-FQ fluorescent probe in the system is cleaved, generating substantial fluorescent signals that can be detected by fluorescent detection equipment (Figure 3B). To obtain the best signal readout during Cas12a-mediated trans-cleavage, we optimized the optimal range of the Cas protein-to-crRNA ratio for detection capability. Maximum fluorescence signal values were obtained using the RAA–CRISPR assay when the Cas protein-to-rRNA molar ratio was 2:1 (Figure 3C). Therefore, we used 200 nM Cas12a and 100 nM crRNA for subsequent experiments.

### 3.3. Effects of Chemical Additives

**Figure 4:**
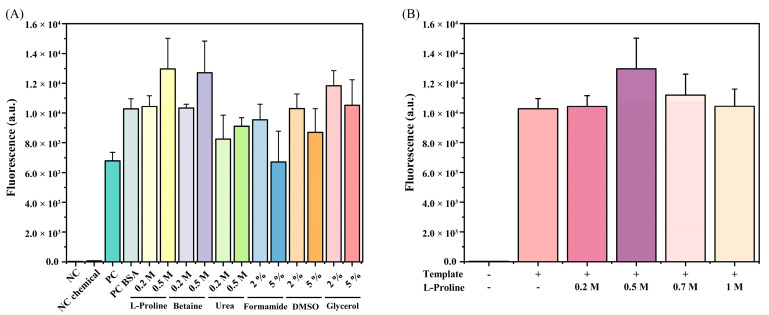
Effects of chemical additives on the CRISPR detection phase. (**A**) Evaluation of the effects of different chemical additives on CRISPR detection systems. NC denotes negative control; NC chemical denotes that no target template was added, but chemical additives were added; PC denotes positive control; PC BSA denotes the addition of target template and BSA. (**B**) The effect of L-proline addition on CRISPR-based detection system.

We further enhanced the fluorescence signal values of the RAA–CRISPR method by adding chemical additives. BSA addition resulted in a significant increase in the endpoint fluorescence signal (Figure 4A), but there was no significant change in the negative control group. Thus, BSA may be an ideal signal enhancer in CRISPR assays. We further investigated whether other chemical additives had a positive synergistic effect. For LbaCas12a-mediated DNA detection, some chemical additives such as L-proline, betaine, and glycerol could enhance fluorescent signals. Among the additives, the addition of L-proline resulted in the most significant enhancement in fluorescence signals. The addition of 0.5 M betaine also enhanced the signal, but by a lesser degree than L-proline. Therefore, L-proline was chosen as the chemical additive to enhance the CRISPR detection system.

To determine the optimal L-proline concentration, a series of concentrations was added (Figure 4B). With increasing final L-proline concentration, the fluorescence signal intensified. The fluorescence signal was highest when the final concentration of L-proline was 0.5 M. Therefore, 0.5 M was the optimal final concentration of L-proline. Thus far, we constructed a CE–RAA–CRISPR detection system by enhancing the endpoint fluorescence signal value of the RAA–CRISPR method through chemical addition.

### 3.4. LOD Comparison of qRAA, RAA–CRISPR, and CE–RAA–CRISPR Methods

**Figure 5:**
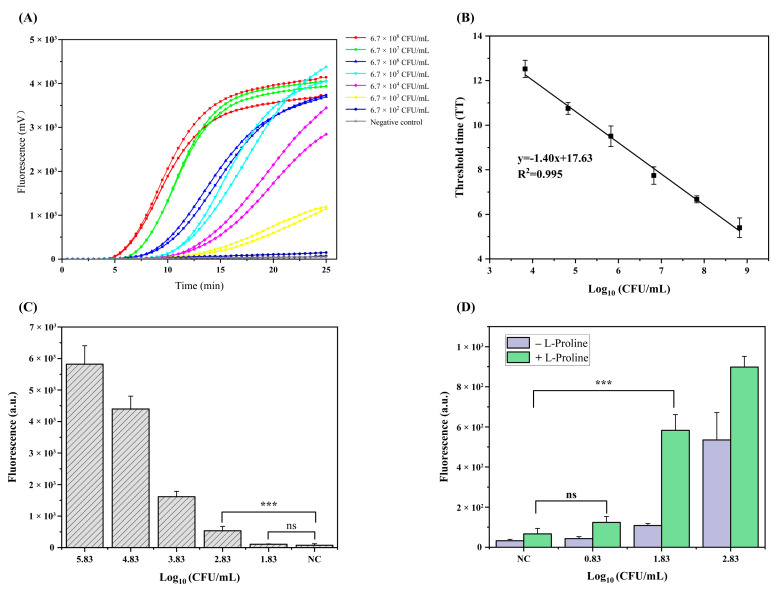
LOD results of different methods, for ten-fold serial dilutions of *V. parahaemolyticus* in pure culture: qRAA (**A**), RAA–CRISPR (**C**), and CE–RAA–CRISPR (**D**). (**B**) Linear relationship between fluorescence intensity and the concentration of *V. parahaemolyticus* over the range of 6.7 × 10^3^ to 6.7 × 10^8^ CFU/mL. *** indicated a statistically significant difference, *p* < 0.001; ns indicates no significant difference.

We compared the LOD of the different quantitative assays. The qRAA and RAA–CRISPR assays were applied to pure cultures with different concentrations of *V. parahaemolyticus*, ranging from 6.7 × 10^1^ to 6.7 × 10^8^ CFU/mL. qRAA detected *V. parahaemolyticus* when the bacterial solution concentration was 6.7 × 10^3^ CFU/mL or higher (Figure 5A). A linear relationship (R^2^ = 0.995) existed between the threshold time and the *V. parahaemolyticus* concentration over the range of 6.7 × 10^3^ to 6.7 × 10^8^ CFU/mL (Figure 5B). Thus, the detection limit of the qRAA method was 6.7 × 10^3^ CFU/mL. In contrast, the lower LOD exhibited by the RAA–CRISPR method (detection limit of 6.7 × 10^2^ CFU/mL) was 10-fold lower than that of the qRAA method (detection limit of 6.7 × 10^3^ CFU/mL; Figure 5C).

Chemical addition further reduced the LOD of the assay. The LOD of CE–RAA–CRISPR was 6.7 × 10^1^ CFU/mL, which was 10 times lower than that of RAA–CRISPR without chemical additives (Figure 5D).

### 3.5. LOD of CE–RAA–CRISPR Method in Artificially Contaminated Shrimp

**Figure 6:**
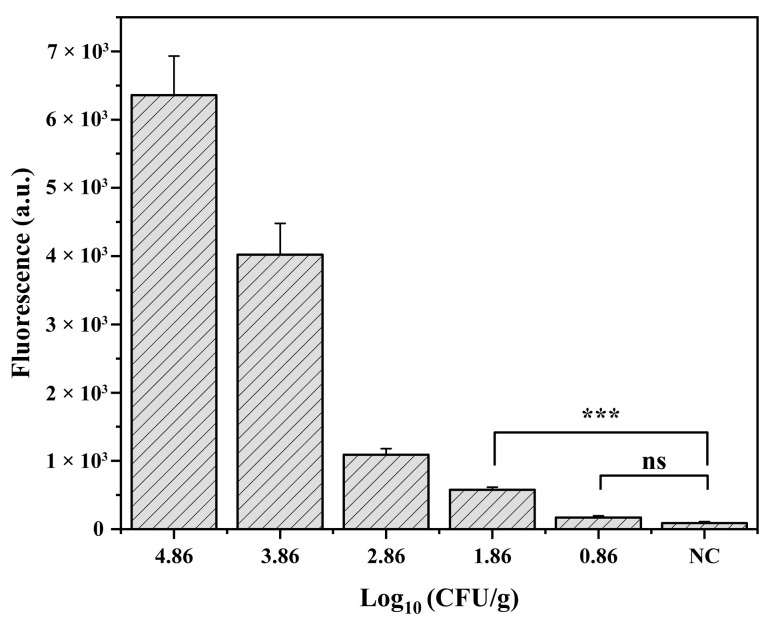
LOD of the CE–RAA–CRISPR for the detection of *V. parahaemolyticus* in shrimp. *** indicated a statistically significant difference, *p* < 0.001; ns indicates no significant difference.

The developed CE–RAA–CRISPR assay was evaluated with shrimp samples spiked with *V. parahaemolyticus* at concentrations of 7.3 × 10^4^ to 7.3 × 10^0^ CFU/g. When the concentration of the bacterial solution in shrimp samples was 7.3 × 10^1^ CFU/g, the fluorescence signal values generated through the CE–RAA–CRISPR method were still significantly different from those for the blank group. The detection limit of the CE–RAA–CRISPR method in artificially contaminated shrimp samples was 7.3 × 10^1^ CFU/g (Figure 6). These results revealed the rapid and sensitive detection advantages of the CE–RAA–CRISPR technique.

### 3.6. Specificity of CE–RAA–CRISPR Assays

In the specificity test, the genome DNA extracted from 22 bacterial strains was used for CE–RAA–CRISPR detection. The reaction was positive only for *V. parahaemolyticus* (both standard and isolated strains), while negative results were obtained for non-*V. parahaemolyticus*, with no cross-reactivity (Table 1). The CE–RAA–CRISPR method exhibited specificity for *V. parahaemolyticus* detection.

### 3.7. Evaluating Consistency between CE–RAA–CRISPR and qPCR Assays Using Actual Samples

**Figure 7:**
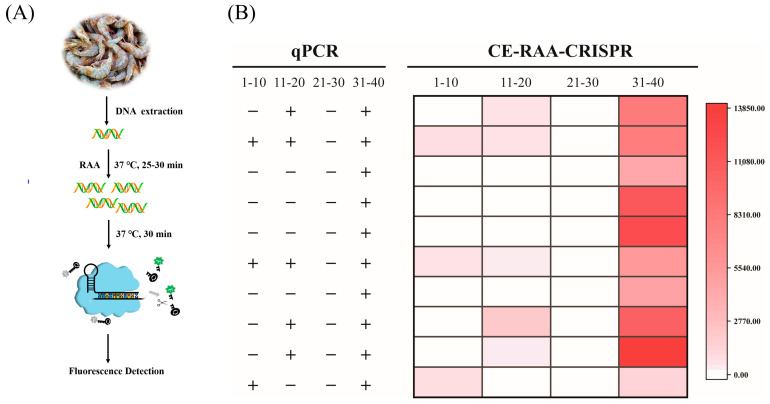
Evaluation of CE–RAA–CRISPR for the detection of *V. parahaemolyticus* in actual food samples. (**A**) Schematic illustration for the detection of *V. parahaemolyticus* in actual food samples using CE–RAA–CRISPR. (**B**) Detection of *V. parahaemolyticus* in 40 aquatic samples using the qPCR method (left) and CE–RAA–CRISPR assay (right).

To evaluate the performance of the CE–RAA–CRISPR assay, 40 actual samples, including shrimp, fish, and clams, were analyzed using both CE–RAA–CRISPR and qPCR assays (Figure 7). In Figure 7B, 1–20 represent the 20 raw shrimp samples, 21–30 represent the 10 fish samples, and 31–40 represent the 10 clam samples. The left side shows the results of qPCR for each sample, and the right side shows the results of the CE–RAA–CRISPR method for each sample. For the CE–RAA–CRISPR test results, we use a heat map to display the results, where the color shades represent the fluorescence value from high and low. Of the 40 samples, 18 tested positive with CE–RAA–CRISPR, which was in 100% diagnostic agreement with the qPCR method. These data and results demonstrated the stable performance of the CE–RAA–CRISPR method on real food matrices and substantiate the practical application of the method for *V. parahaemolyticus* detection.

## 4. Discussion

*V. parahaemolyticus*, a pathogenic bacterium, has caused huge economic losses in both the food and mariculture industries. Therefore, the rapid, accurate, and sensitive detection of *V. parahaemolyticus* is essential for the effective control and prevention of its outbreak and spread [26]. Current molecular diagnostic techniques for *V. parahaemolyticus* mainly rely on PCR-based techniques, such as qPCR and ddPCR. Although these techniques demonstrate strong applicability, they require expensive instruments and professional expertise [27]. Therefore, strategies for the convenient and rapid detection of *V. parahaemolyticus* are urgently needed.

The CRISPR/Cas12a system further enhances nucleic acid detection. Although traditional methods based on CRISPR systems, such as HOLMES [28] and DETECTR [29], have been successfully applied in nucleic acid detection, they usually require the transfer of recombinase polymerase amplification or PCR amplification products, which increases operational complexity and the risk of sample cross-contamination [20,30]. In our study, CE–RAA–CRISPR, a convenient and highly sensitive method for the rapid one-pot detection of *V. parahaemolyticus*, was established.

Primer and probe sequences are the main components of nucleic-acid-based detection systems. It is necessary to screen the best primer sets to develop more rapid and effective detection methods. In this study, all reverse primers were screened against a single forward primer, and the best reverse primer was selected and then used to screen all of the forward primers to find a good primer pair. This optimization approach is quicker and more convenient for finding a primer pair. RAA primers are longer than PCR primers. The RAA primer used in this study was 33 nucleotides in length; however, shorter qPCR primers have also been useful in RAA. Therefore, the primers can be adjusted and optimized to meet the specific needs of the experiment [31].

Several factors, such as the Cas/crRNA ratio and reporter groups, affect the ability of the CRISPR system to detect nucleic acids. Optimization of the Cas/crRNA ratio revealed that the maximum fluorescence signal was obtained through the RAA–CRISPR method when the Cas protein-to-crRNA molar ratio was 2:1. In the experiment performed by Yin et al. [32], this concentration was less effective for enhancing the fluorescence signal, presumably because the one-pot detection method introduces more amplification products. As reported by several authors [20,30,33], the optimal Cas/crRNA ratio varies among targets or detection systems. Li et al. [25] demonstrated the importance of the Cas/crRNA stoichiometry, as the presence of excess crRNA reduces the fluorescence signal. This was also demonstrated in the present study, where the presence of excess Cas protein and crRNA reduced the fluorescence signal. Other studies have reported similar results [33].

Several strategies for improving the sensitivity of CRISPR-based nucleic acid detection have been explored, such as reaction temperature and time optimization, crRNA modification, reporter probe length optimization, and chemical reagent addition. In this study, BSA and L-proline were added to the CRISPR assay reaction to lower the LOD of the method. The method had a detection limit of 67 CFU/mL in pure culture and 73 CFU/g in shrimp. It was more sensitive than previously reported methods, which exhibited a sensitivity of 1.35 × 10^3^ CFU/mL for *Listeria monocytogenes* in grass carp [33], 5.4 × 10^2^ CFU/mL for *Staphylococcus aureus* in pure culture [34], and 2 × 10^3^ CFU/mL for *Salmonella* in powered infant formula milk [35]. It was reported that the LOD of the method using only real-time RPA for *V. parahaemolyticus* detection was 1.02 × 10^2^ copies/reaction, but for artificially contaminated samples with different bacteria concentrations, the LODs were 4, 1, and 7 CFU/25 g in oyster sauce, codfish and sleeve-fish, respectively, after enrichment for 6 h [36]. In contrast, the method established in this study has a lower detection limit without pre-enrichment, and the method not only has a lower detection limit but also a shorter detection time. Furthermore, the other commonly detected pathogenic bacteria were all negative, and the *V. parahaemolyticus* reference strain and environmental isolates were detected using the method established in this study. Therefore, the proposed CE–RAA–CRISPR method provides sufficient sensitivity and specificity for *V. parahaemolyticus* detection.

Compared with traditional PCR-based methods, the proposed CRISPR-based assay exhibited cleavage activity on the target DNA, indicating that it can reduce cross-contamination in the laboratory. Moreover, the amplification condition of 37 °C also reduced the loss and contamination caused by high temperatures during PCR [37]. In addition, the PCR-based methods required more than 1.5 h to complete, while the CE–RAA–CRISPR method provided results in less than 1 h. Application of the assay to real food samples showed accurate and consistent detection results compared with qPCR.

In summary, we developed CE–RAA–CRISPR, a convenient and sensitive method for *V. parahaemolyticus* detection. The use of RAA not only reduces the inspection time but also avoids the use of a complex thermal cycler [14]. The CRISPR detection stage is improved by chemical additives. The results suggest that this platform can meet the demand for *V. parahaemolyticus* detection and is important to food safety supervision.

## Figures and Tables

**Table 1:** Bacterial strains used for the evaluation of the specificity of CE–RAA–CRISPR in this study.

Serial Number	Species	Strain	CE–RAA–CRISPR Results
1	*Vibrio parahaemolyticus*	ATCC 17802	+
2	*Vibrio parahaemolyticus*	JNUFN 01	+
3	*Vibrio parahaemolyticus*	JNUFN 02	+
4	*Vibrio parahaemolyticus*	JNUFN 03	+
5	*Vibrio parahaemolyticus*	JNUFN 04	+
6	*Vibrio parahaemolyticus*	JNUFN 05	+
7	*Vibrio parahaemolyticus*	JNUFN 06	+
8	*Vibrio parahaemolyticus*	JNUFN 07	+
9	*Vibrio parahaemolyticus*	JNUFN 08	+
10	*Vibrio parahaemolyticus*	JNUFN 09	+
11	*Vibrio parahaemolyticus*	JNUFN 10	+
12	*Escherichia coli* O157:H7	ATCC 35150	−
13	*Enteroinvasive Escherichia coli*	CICC 10662	−
14	*Enterotoxigenic Escherichia coli*	CICC 10667	−
15	*Escherichia coli* O127: K63	CICC 10411	−
16	*Escherichia coli* EPEC O86: K61	CICC 10412	−
17	*Staphylococcus aureus*	ATCC 25923	−
18	*Cronobacter sakazakii*	ATCC 29544	−
19	*Listeria monocytogenes*	ATCC 19115	−
20	*Vibrio alginolyticus*	ATCC 33787	−
21	*Pseudomonas aeruginosa*	ATCC 15442	−
22	*Pseudomonas aeruginosa*	ATCC 27853	

ATCC, American Type Culture Collection, USA; JNUFN, Institute of Food Safety and Nutrition of Jinan University; CICC, China Center of Industrial Culture Collection; + and − indicate positive and negative reaction, respectively.

**Table 2:** Primers and probe used in this study.

Name	Sequence (5′-3′)
RAA-F1	AGATTTGGCGAACGAGAACGCAGACATTACG
RAA-F2	TTAGATTTGGCGAACGAGAACGCAGACATTA
RAA-F3	TTAGATTTGGCGAACGAGAACGCAGACATTACG
RAA-R1	GTCACCGAGTGCAACCACTTTGTTGATTTGA
RAA-R2	TTGCCTGTATCAGACAAGCTGTCACCGAGTG
RAA-R3	TGTTGCCTGTATCAGACAAGCTGTCACCGAGTG
RAA-Probe	TGACAATCGCTTCTCATACAACCACACGA/i6FAMdT//THF//iBHQ1dT/GGAGCAACGACGCA
ssDNA-FQ reporter	6-FMA-TTATT-BHQ1
crRNA	UAAUUUCUACUAAGUGUAGAUAUCUGGCUGCAUUGCUGCGU
qPCR-F	GTTCATCAAGGCACAAGCGA
qPCR-R	ACAGACGATGAGCGGTTGAT
qPCR-P	FAM-CGTTGTTTGATACTCACGCCTTGTTCG-BHQ

The element of the crRNA underlined is nucleotide sequences complementary to the template strand.

## Data Availability

The data presented in this study are available on request from the corresponding author.
